# Non-Mature miRNA-Encoded Micropeptide miPEP166c Stimulates Anthocyanin and Proanthocyanidin Synthesis in Grape Berry Cells

**DOI:** 10.3390/ijms25031539

**Published:** 2024-01-26

**Authors:** Mariana Vale, Hélder Badim, Hernâni Gerós, Artur Conde

**Affiliations:** 1 Centre of Molecular and Environmental Biology (CBMA), Department of Biology, University of Minho, 4710-057 Braga, Portugal; marianasantosvale@gmail.com (M.V.); helderbadim@hotmail.com (H.B.); arturconde@bio.uminho.pt (A.C.); 2 Centre of Biological Engineering (CEB), Department of Engineering, University of Minho, 4710-057 Braga, Portugal

**Keywords:** non-mature miRNA-encoded micropeptides (miPEPs), secondary metabolism, anthocyanins, proanthocyanidins, grape berry cells

## Abstract

The phenylpropanoid and flavonoid pathways exhibit intricate regulation, not only influenced by environmental factors and a complex network of transcription factors but also by post-transcriptional regulation, such as silencing by microRNAs and miRNA-encoded micropeptides (miPEPs). VviMYBC2-L1 serves as a transcriptional repressor for flavonoids, playing a crucial role in coordinating the synthesis of anthocyanin and proanthocyanidin. It works in tandem with their respective transcriptional activators, VviMYBA1/2 and VviMYBPA1, to maintain an equilibrium of flavonoids. We have discovered a miPEP encoded by miR166c that appears to target *VviMYBC2-L1*. We conducted experiments to test the hypothesis that silencing this transcriptional repressor through miPEP166c would stimulate the synthesis of anthocyanins and proanthocyanidins. Our transcriptional analyses by qPCR revealed that the application of exogenous miPEP166c to Gamay Fréaux grape berry cells resulted in a significant upregulation in flavonoid transcriptional activators (VviMYBA1/2 and VviMYBPA1) and structural flavonoid genes (*VviLDOX* and *VviDFR*), as well as genes involved in the synthesis of proanthocyanidins (*VviLAR1* and *VviANR*) and anthocyanins (*VviUFGT1*). These findings were supported by the increased enzyme activities of the key enzymes UFGT, LAR, and ANR, which were 2-fold, 14-fold, and 3-fold higher, respectively, in the miPEP166c-treated cells. Ultimately, these changes led to an elevated total content of anthocyanins and proanthocyanidins.

## 1. Introduction

The quality and yield of grape berries are greatly influenced by the adaptability of grapevines (*Vitis vinifera* L.) to their environment. A crucial aspect of grape berry development is their secondary metabolism, which plays a vital role in determining the quality characteristics of both the berries and the resulting wine. Phenolic compounds, the most abundant group of secondary metabolites in grapevines, contribute significantly to these traits [1,2]. Beyond their impacts on the color, aroma, texture, and stability of the berries and wine, phenolic compounds also possess notable antioxidant properties. To enhance the composition of the phenolic compounds of interest, it is crucial to gain a comprehensive understanding of the intricate and interconnected pathways, such as phenylpropanoids and flavonoids. By comprehending their functioning, we can effectively modulate these pathways. In addition to external environmental factors, intrinsic molecular mechanisms, including transcription factors (TFs), play a pivotal role in regulating these pathways. TFs can act independently or in conjunction with other TFs, coordinating their efforts to achieve different metabolic outcomes based on the plant’s requirements or in response to environmental stress [2].

A critical component of this coordinated regulatory network is the ternary MBW transcription complex, which consists of a basic helix-loop-helix (bHLH) protein, a WD repeat protein (WDR), and a MYB transcription factor [3]. While all three components are necessary to activate anthocyanin biosynthesis, it is the MYB transcription factor that confers specificity to this complex. It determines the patterns and spatial localization of anthocyanin biosynthesis [4]. Therefore, the MYB transcription factor plays a crucial role in regulating the production and distribution of anthocyanins. In grapevines, various transcription factors have been identified as positive regulators (activators) or repressors of specific metabolic pathways. For instance, the anthocyanin-related TFs MYBA1/A2 and the proanthocyanidin-related TFs MYBPA1/A2 function as activators [4,5]. Conversely, the R3-MYB and R2R3-MYB repressors play a negative regulatory role in anthocyanin synthesis. R3-MYB factors compete with activators for binding to bHLH transcription factors, preventing the formation of the MBW activation complex [6]. The R2R3-MYB repressors also interact with bHLH factors but possess a distinctive C2 repressor motif clade and an ethylene response factor (ERF)-associated amphiphilic repression (EAR) motif, which are involved in transcriptional repression [7]. Therefore, the regulation of anthocyanin biosynthesis involves a coordinated interplay between transcriptional activators and repressors.

In grapevines, the R2R3-MYB transcriptional repressors, including MYBC2-L1, MYBC2-L2, MYBC2-L3, and MYB4-like, have been shown to finely modulate different branches of the phenylpropanoid pathway in petunia plants. However, only MYBC2-L1 negatively impacts both anthocyanin and proanthocyanidin synthesis in grapevines [7]. MYBC2-L1 was previously identified as a repressor of proanthocyanidin-related genes (*LAR* and *ANR*) by interacting with the transcriptional activator MYBPA1 to maintain a balance in proanthocyanidin content [8]. Recent studies by Xie et al. [9] confirmed that MYBA1 (an anthocyanin transcriptional activator) and MYBC2-L1 (a repressor) function in a coordinated manner, providing regulatory feedback for the cell-specific production of anthocyanins in grape berries, although the underlying mechanism is not yet fully understood. Additionally, studies in forage legumes suggest that MYBC2-L1 can affect anthocyanin synthesis directly by repressing structural genes (*LDOX*, *DFR*, or *UFGT1*) or indirectly by downregulating the expression of MYBA1 or bHLH activator genes [3]. Interestingly, despite being a repressor, *MYBC2-L1* expression has been observed to correlate with the expression of *MYBA1* and anthocyanin accumulation, indicating a complex regulatory relationship.

The regulation of the flavonoid pathway not only involves the complex network of transcription factors but also post-transcriptional regulation mediated by microRNAs and miRNA-encoded micropeptides (miPEPs) [10]. These miPEPs enhance the transcription and accumulation of the corresponding primary miRNA, leading to an intensified downregulation of the targeted genes through a positive feedback loop [11]. Although miPEPs are a recent discovery in plants, a few have been described in various species. For example, miPEP171b promotes lateral root development in *Medicago truncatula* [11], while the exogenous application of miPEP172c enhances nodule formation and reduces the need for nitrogen fertilization in *Glycine max* [12]. In Arabidopsis, miPEP858 modulates the expression of the target genes involved in plant growth, development, and the phenylpropanoid pathway by inducing the expression of miR858 [13]. In grapevines, a miPEP called miPEP171d1 has been identified and described as promoting root organ development and enhancing adventitious root formation. A promising feature in addressing one of the biggest challenges in clonal propagation of *Vitis vinifera* is the difficulty of the critical step of rooting [14].

In a previous study, we demonstrated that miPEP164c negatively regulates the synthesis of proanthocyanidins and simultaneously stimulates anthocyanin synthesis by targeting the corresponding miRNA (*miR164c*) and repressing the *VvMYBPA1*, *VviLAR1*, and *VviANR* genes [15]. The inhibition of the proanthocyanidin pathway leads to the upregulation of the anthocyanin pathway, as both pathways compete for the same substrate. This finding highlighted the potential of exogenously applied miPEPs in modulating secondary metabolic pathways and increasing the synthesis of secondary metabolites, such as anthocyanins, crucial for grape quality and possessing bioactive properties. Based on these observations, we identified miPEP166c in silico, which potentially enhances the synthesis of anthocyanins and proanthocyanidins by targeting the transcriptional repressor VviMYBC2-L1 via its corresponding miRNA, *miR166c*. Therefore, in this study, we aimed to investigate whether the exogenous application of miPEP166c could increase the synthesis and accumulation of anthocyanins and proanthocyanidins by inhibiting VviMYBC2-L1.

## 2. Results

### 2.1. Identification and In Silico Analysis of the Grapevine Micropeptide miPEP166c

An in silico examination, aimed at identifying micropeptides through the screening of small open reading frames (sORFs), led to the discovery of a potential micropeptide encoded in the primary sequence of miR166c (pri-miR166c). In silico predictions indicated that miR166c could post-transcriptionally inhibit the grapevine transcription factor VviMYBC2-L1, a recognized repressor of flavonoid-related transcriptional activators. Specifically, VviMYBC2-L1 is known to suppress the proanthocyanidin-related transcription factor VviMYBPA1, which acts through the activation of *VviLAR1* and *VviANR*, as well as the anthocyanin-related transcription factor VviMYBA1/2, which functions via the activation of *VviUFGT1*. Table 1 provides comprehensive details obtained through the in silico analysis concerning the selected miPEP for this study, encompassing its amino acid sequence, assigned name, mature miRNA name, miRbase accession number, and that of its precursor miRNA (pre-miRNA).

### 2.2. Transcriptional and Biochemical Modifications Induced by miPEP166c on the Precursor form of miR166c

**Figure 1:**
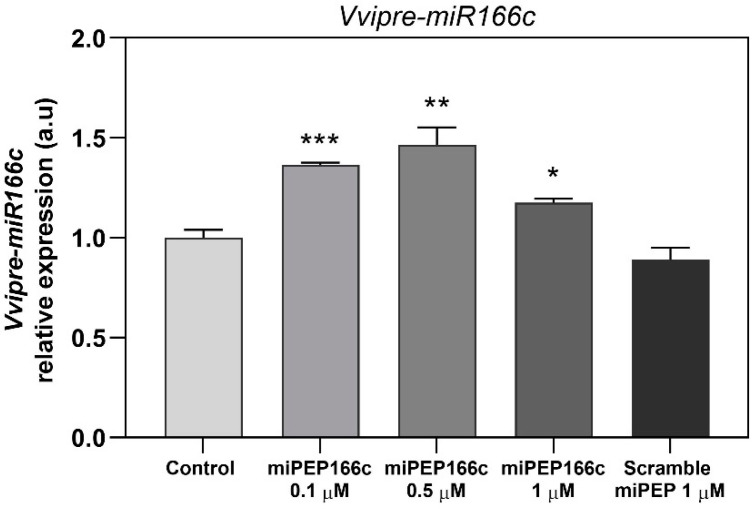
Steady-state transcript levels of *Vvipre-miR166c* in suspension-cultured grape berry cells (cv. Gamay) following a 10-day elicitation with varying concentrations of miPEP166c. The gene expression analysis, conducted through real-time qPCR, was normalized with the expression of the reference genes *VviACT1* and *VviGAPDH*. The values are presented as the mean ± SD of three biological replicates analyzed in three different experiments. Asterisks in the figure indicate statistical significance based on the Student’s *t*-test (* *p* < 0.05, ** *p* < 0.01, and *** *p* < 0.001, *n* = 18).

To explore the potential regulatory influence of miPEP166c on enhancing the synthesis of its corresponding mature miRNA, *miR166c*, we assessed the transcript levels of its non-mature form, *pre-miR166c*, across various concentrations of miPEP166c, as shown in Figure 1. The results indicate a notable and statistically significant stimulation of pre-miR166c expression at all concentrations of miPEP166c tested. Specifically, there was a 36% increase in the cells treated with 0.1 µM, a 46% increase in the cells treated with 0.5 µM, and an 18% increase in the cells treated with 1 µM of miPEP166c, as shown in Figure 1. In contrast, treatment with the scrambled miPEP at the maximum concentration did not induce any changes in pre-miR166c expression.

### 2.3. The Impact of Exogenous Application of miPEP166c on Key Secondary Metabolites in Grape Berry Cells

**Figure 2:**
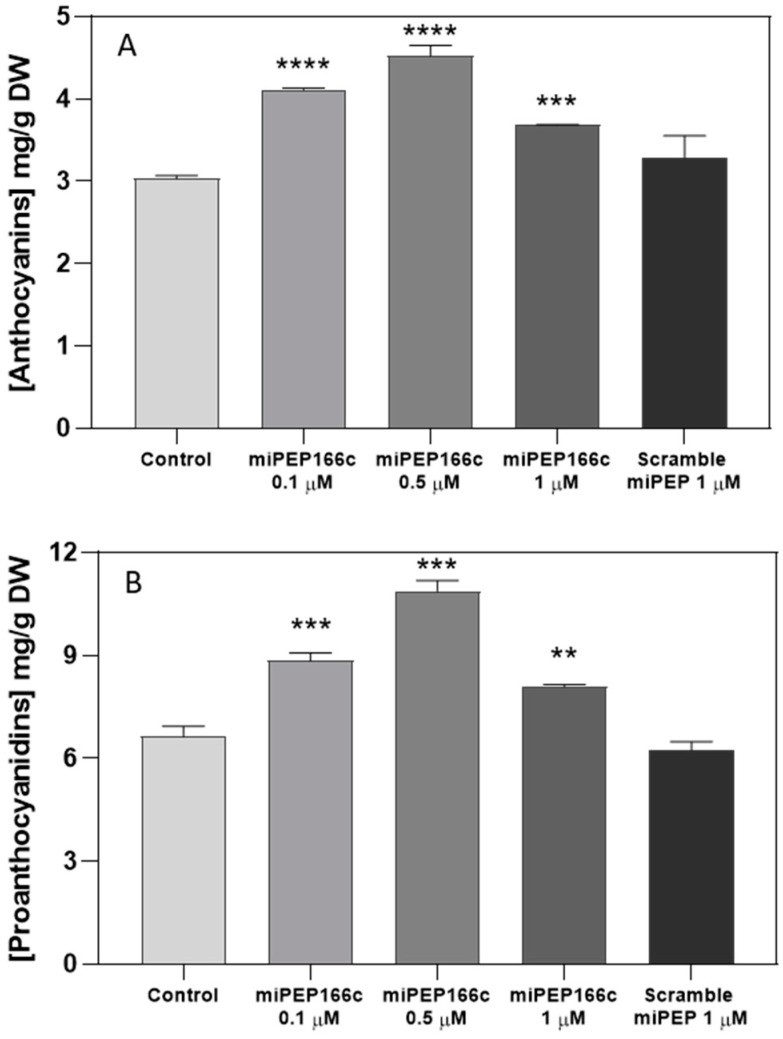
The effect of the exogenous application of different concentrations of miPEP166c on total anthocyanin content (**A**) and on total proanthocyanidin content (**B**) in suspension-cultured grape berry cells (cv. Gamay) 10 d after elicitation with miPEP166c. Anthocyanin concentration is represented as mg of cyanidin 3-glucoside (C-3-G) equivalents per g of fresh weight (DW). The values are presented as the mean ± SD of three biological replicates analyzed in three different experiments. Asterisks indicates statistical significance in relation to the control (Student’s *t*-test; ** *p* < 0.01; *** *p* < 0.001; and **** *p* < 0.0001; *n* = 18).

The quantitative analysis of grape berry secondary metabolites through spectrophotometry revealed a noteworthy increase in anthocyanin content under miPEP166c treatment. The highest concentration of anthocyanins was observed in the cells treated with 0.5 µM of miPEP166c, exhibiting a significant 49% increase. This resulted in a content of 4.5 mg of cyanidin-3-glucoside equivalents per gram of dry cell weight, in contrast to the 3 mg per gram of dry weight observed in control cells (Figure 2A).

Simultaneously, proanthocyanidin content displayed a significant stimulation across all concentrations tested, with the highest content recorded in the cells treated with 0.5 µM of miPEP166c. In this case, the proanthocyanidin content reached 10.9 mg of (+)-catechin equivalents per gram of dry weight, marking a substantial 64% increase compared to the 6.6 mg per gram of dry weight observed in control cells (Figure 2B). It is noteworthy that the exogenous application of a scrambled miPEP at the highest concentration of 1 µM did not induce any changes in the contents of both anthocyanins and proanthocyanidins.

### 2.4. Transcriptional Changes Induced by miPEP166c on Its Putative Target VviMYBC2-L1

**Figure 3:**
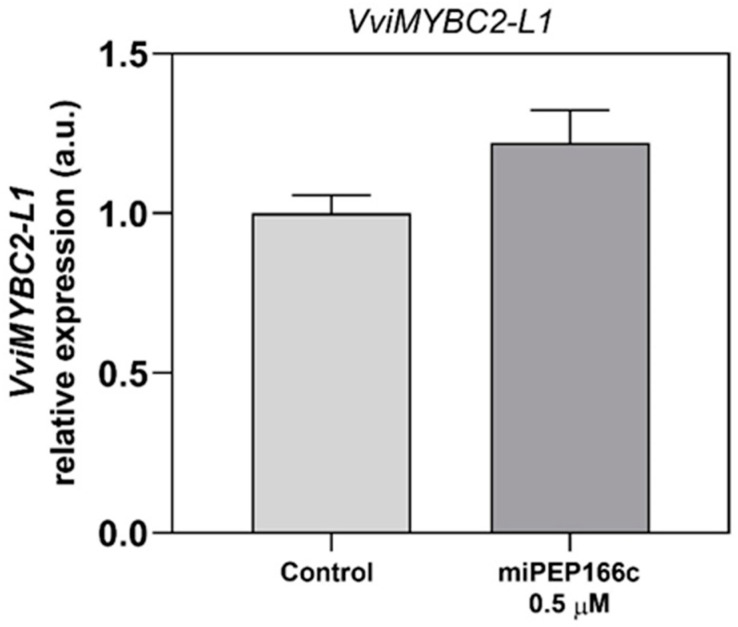
Steady-state transcript levels of *VviMYBC2-L1* in suspension-cultured grape berry cells (cv. Gamay) after 10 days of elicitation with 0.5 µM of miPEP166c. The gene expression analysis, conducted through real-time qPCR, was normalized with the expression of the reference genes *VviACT1* and *VviGAPDH*. The values are presented as the mean ± SD of three biological replicates analyzed in three different experiments.

The expression analysis of VviMYBC2-L1, the putative target of miPEP166c and the gene encoding for the proanthocyanidin (PA) and anthocyanin-related transcriptional repressor, revealed that its expression was not significantly affected under miPEP166c treatment, as shown in Figure 3.

### 2.5. Transcriptional and Biochemical Alterations Induced by miPEP166c on the Branch Responsible for Proanthocyanidin Synthesis

**Figure 4:**
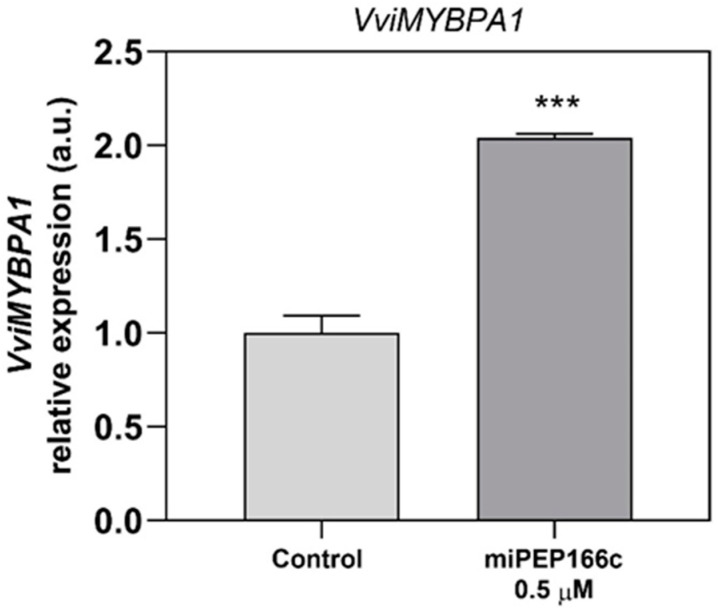
Steady-state transcript levels of *VviMYBPA1* in suspension-cultured grape berry cells (cv. Gamay) after 10 days of elicitation with 0.5 µM of miPEP166c. The gene expression analysis, performed through real-time qPCR, was normalized using the expression of the reference genes *VviACT1* and *VviGAPDH*. The values are presented as the mean ± SD of three biological replicates analyzed in three different experiments. Asterisks denote statistical significance, as determined using the Student’s *t*-test (*** *p* < 0.001; *n* = 18).

The proanthocyanidin-related transcription factor VviMYBPA1’s transcript levels were upregulated in Gamay cells elicited with miPEP166c with an increase of 2-fold when compared to the control cells (Figure 4).

**Figure 5:**
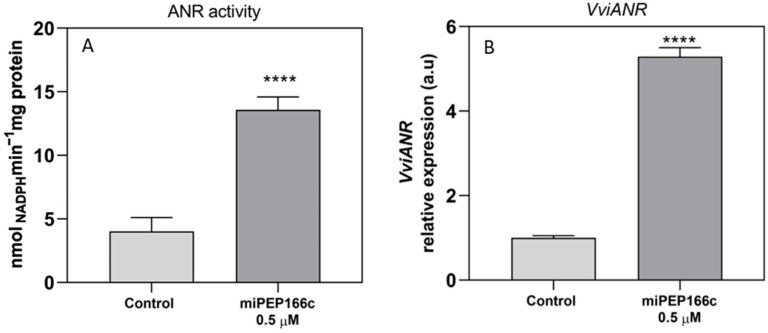
The impact on the specific activity of ANR (**A**) and the steady-state transcript levels of *VviANR* (**B**) in suspension-cultured grape berry cells (cv. Gamay) is illustrated 10 days after elicitation with 0.5 µM of miPEP166c. The gene expression analysis, conducted through real-time qPCR, was normalized with the expression of the reference genes *VviACT1* and *VviGAPDH*. The values are presented as the mean ± SD of three biological replicates analyzed in three different experiments. Asterisks denote statistical significance, as determined using the Student’s *t*-test; (**** *p* < 0.0001; *n* = 18). Additionally, the ANR biochemical activity, represented as *V*_max_ in grape berry cells under miPEP166c treatment, is also depicted. The values are presented as the mean ± SD of three biological replicates, with the asterisks indicating statistical significance determined using the Student’s *t*-test (**** *p* < 0.0001; *n* = 18).

The specific activity of ANR was significantly increased by 3-fold, reaching a maximum velocity (*V*_max_) of 13.5 nmol min^−1^ mg protein^−1^ (Figure 5A). In accordance with the biochemical changes, the expression levels of *VviANR* were also stimulated with a significant increase of 5-fold in the cells under miPEP166c treatment when compared to the control cells, as shown in Figure 5B.

**Figure 6:**
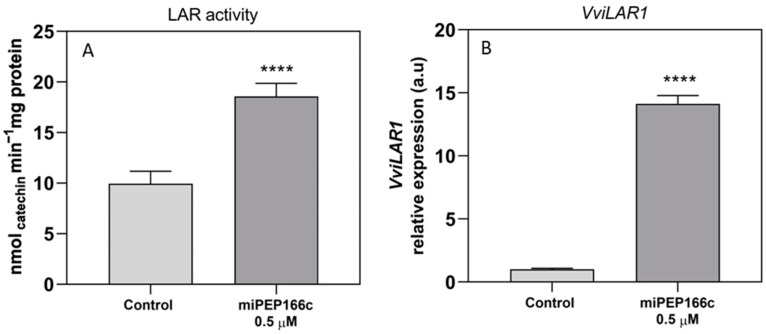
The impact on the specific activity of LAR (**A**) and the steady-state transcript levels of *VviLAR1* (**B**) in suspension-cultured grape berry cells (cv. Gamay) is demonstrated 10 days after elicitation with 0.5 µM of miPEP166c. Gene expression analysis, performed using real-time qPCR, was normalized with the expression of the reference genes *VviACT1* and *VviGAPDH*. The values are presented as the mean ± SD of three biological replicates analyzed in three different experiments. Asterisks denote statistical significance, as determined using the Student’s *t*-test; (**** *p* < 0.0001; *n* = 18). Additionally, the LAR biochemical activity, represented as *V*_max_ in grape berry cells under miPEP166c treatment, is also depicted. The values are presented as the mean ± SD of three biological replicates analyzed in three different experiments, with the asterisks indicating statistical significance determined using the Student’s *t*-test (**** *p* < 0.0001; *n* = 18).

The specific activity of LAR had a significant increase of 86% in the cells treated with miPEP166c, reaching a *V*_max_ of 18.5 nmol min^−1^ mg protein^−1^ (Figure 6A). The transcript levels of *VviLAR1* were also upregulated, with a very significant increase of 14-fold (Figure 6B). 

### 2.6. Transcriptional and Biochemical Alterations Induced by miPEP166c on the Branch Responsible for Anthocyanin Synthesis

**Figure 7:**
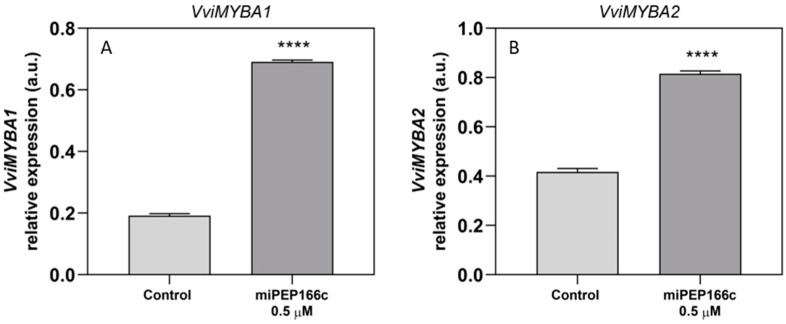
Steady-state transcript levels of *VviMYBA1* (**A**) and *VviMYBA2* (**B**) in suspension-cultured grape berry cells (cv. Gamay) are depicted 10 days after elicitation with 0.5 µM of miPEP166c. Gene expression analysis, conducted through real-time qPCR, was normalized with the expression of the reference genes *VviACT1* and *VviGAPDH*. The values are presented as the mean ± SD of three biological replicates analyzed in three different experiments. Asterisks denote statistical significance, as determined using the Student’s *t*-test; (**** *p* < 0.000; *n* = 18).

The expression of *VviMYBA1*, a gene encoding for one of the transcriptional factors responsible for anthocyanin biosynthesis activation via *VviUFGT1* upregulation, was stimulated under miPEP166c treatment, with an increase of 4-fold in the abundance of transcripts observed (Figure 7A). *VviMYBA2* transcript levels were also analyzed and showed a significant increase of 95% in the cells treated with miPEP166c (Figure 7B).

**Figure 8:**
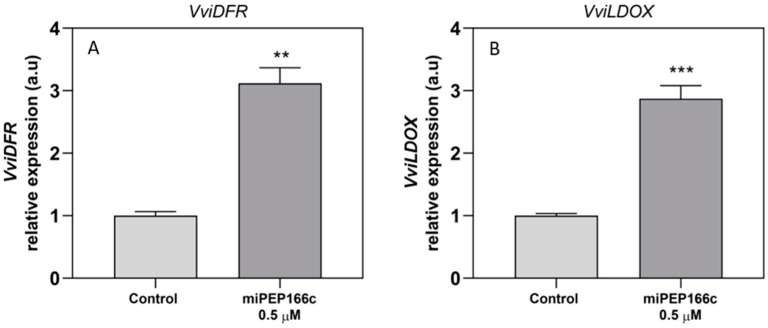
Steady-state transcript levels of *VviDFR* (**A**) and *VviLDOX* (**B**) in suspension-cultured grape berry cells (cv. Gamay) are presented 10 days after elicitation with 0.5 µM of miPEP166c. Gene expression analysis, performed using real-time qPCR, was normalized with the expression of the reference genes *VviACT1* and *VviGAPDH*. The values are expressed as the mean ± SD of three biological replicates analyzed in three different experiments. Asterisks signify statistical significance, as determined using the Student’s *t*-test; (** *p* < 0.01; *** *p* < 0.001; *n* = 18).

The transcript levels of *VviDFR* were upregulated under miPEP166c treatment, with a significant increase of 3-fold (Figure 8A). *VviLDOX* was also significantly stimulated, with an increase of 3-fold when compared to the control cells (Figure 8B).

**Figure 9:**
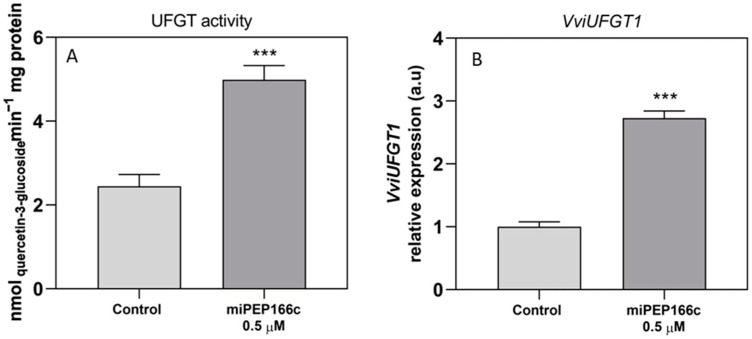
The effect on the specific activity of UFGT (**A**) and the steady-state transcript levels of *VviUFGT1* (**B**) in suspension-cultured grape berry cells (cv. Gamay) is depicted 10 days after elicitation with 0.5 µM of miPEP166c. Gene expression analysis, conducted through real-time qPCR, was normalized with the expression of the reference genes *VviACT1* and *VviGAPDH*. The values are presented as the mean ± SD of three biological replicates analyzed in three different experiments. Asterisks indicate statistical significance, as determined using the Student’s *t*-test; (*** *p* < 0.001; *n* = 18). The UFGT biochemical activity is represented as the *V*_max_ in grape berry cells under miPEP164c treatment. Values are the mean ± SD of three biological replicates analyzed in three different experiments. Asterisks indicate statistical significance (Student’s *t*-test; *** *p* < 0.001; *n* = 18).

The activity of the UFGT enzyme was upregulated by a significant increase of 2-fold, reaching a *V*_max_ of 5 nmol min^−1^ mg protein^−1^ (Figure 9A). The transcript levels of *VviUFGT1* were also stimulated with a very significant increase of 3-fold (Figure 9B).

**Figure 10:**
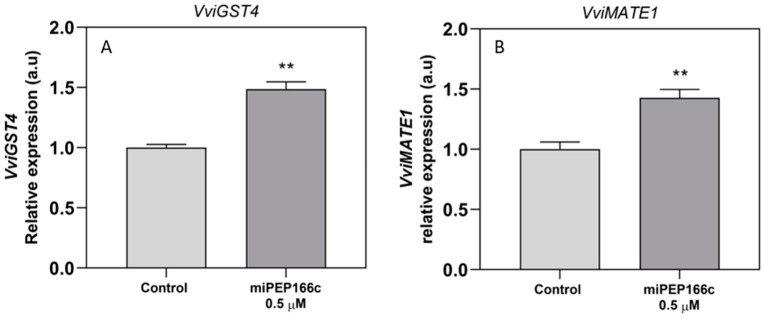
Steady-state transcript levels of *VviGST4* (**A**) and *VviMATE1* (**B**) in suspension-cultured grape berry cells (cv. Gamay) 10 days after elicitation with 0.5 µM of miPEP166c. The gene expression analysis, conducted through real-time qPCR, was normalized with the expression of the reference genes *VviACT1* and *VviGAPDH*. The values are presented as the mean ± SD of three biological replicates analyzed in three different experiments. Asterisks indicate statistical significance, as determined using the Student’s *t*-test; (** *p* < 0.01; *n* = 18).

*VviGST4*, encoding for a protein that stabilizes anthocyanins before their transport into the vacuole, was revealed to be significantly upregulated, with an increase of 49% compared to the control cells (Figure 10A). The same was observed with *VviMATE1*, responsible for anthocyanin accumulation in the vacuole, with the results showing an increase of 43% in miPEP166c-treated cells (Figure 10B). 

## 3. Discussion

Despite being a relatively recent discovery, miPEPs have emerged as a promising avenue for plant gene regulation. Their unique ability to exhibit specificity towards their associated miRNAs makes them particularly valuable, offering a swift and transient response to adverse abiotic conditions or the modulation of specific quality-related traits in plants [16]. This distinctive feature of miPEPs circumvents the need for laborious and costly genetic transformations in crops due to their being a relatively easy-to-implement technology in which many physiological phenotypes of agronomical interest can be achieved by simply applying a specific miPEP [16]. Extending their influence across various plant developmental processes, miPEPs present an opportunity to enhance agronomically important characteristics. In addition to their recognized potential as a valuable agronomic tool, miPEPs can be explored in vitro within a biotechnological context. Their ease of use and relatively cost-effective modulation of secondary metabolic pathways can result in the overproduction of various bioactive compounds possessing nutraceutical characteristics. These compounds hold significant interest for diverse industries, including pharmacology and cosmetics. In the present study, we aimed to validate the application of miPEP166c in suspension-cultured grape berry cells of Gamay Fréaux to effectively modulate secondary metabolic pathways to increase the synthesis of specific metabolites of interest, notably anthocyanins and proanthocyanidins. These compounds play a pivotal role in shaping the quality of grape berries and, consequently, contribute significantly to the quality of wines [9].

### 3.1. The Exogenous Application of miPEP166c Promotes the Accumulation of miR166c and Modifies the Dynamics of VviMYBC2-L1–VviMYBA1/2 Interactions in the Regulation of PAs and Anthocyanin Synthesis

The outcomes of our study revealed that supplementing Gamay cv. grape berry cell suspensions with exogenous miPEP166c led to an augmented accumulation of its corresponding pre-miR166c. Notably, all tested concentrations of miPEP166c elicited a substantial upregulation in the abundance of pre-miR166c transcripts, representing the stem–loop form of miR166c. The most pronounced expression was observed at 0.5 µM of miPEP166c, resulting in a noteworthy 46% increase, as shown in Figure 1.

VviMYBC2-L1 was initially characterized as a transcriptional repressor of proanthocyanidins (PAs) through its competitive interaction with the well-established transcriptional activator VviMYBPA1 [8]. However, its involvement in the anthocyanin biosynthetic pathway was elucidated by Cavallini et al. [7]. Unlike VviMYBC2-L2 and VviMYBC2-L3, which exhibited a strong correlation with PA-related genes primarily during the early stages of grape berry development, VviMYBC2-L1 demonstrated a unique ability to negatively regulate anthocyanin synthesis [6,7]. This effect was attributed to VviMYBC2-L1′s capacity to interact with other basic helix-loop-helix (bHLH) factors, competing with anthocyanin transcriptional activators for DNA-binding sites. Additionally, it directly repressed *VviUFGT1*, further contributing to its role in anthocyanin downregulation [7].

In their work with Yan73 berries, a teinturier variety, Xie et al. [5] observed that although VviMYBC2-L1 acts as a transcriptional repressor of anthocyanins, its expression pattern correlates with that of the transcriptional activator VviMYBA1 and the accumulation of anthocyanins. Similar expression profiles were noted in other species, such as the strawberry *FaMYB112* and the peach *PpMYB18*, where the expression of anthocyanin repressors peaked during the ripening stage or fruit development stages associated with anthocyanin and proanthocyanidin synthesis [17,18]. This apparent discrepancy in expression patterns and anthocyanin accumulation prompted the proposal of a negative feedback loop controlling anthocyanin biosynthesis: activators activate repressors, repressors repress activators, and repressors repress repressors. The conclusion drawn was that VviMYBA1 (transcriptional activator) and VviMYBC2-L1 (transcriptional repressor) coordinate to regulate Yan73 berry’s flesh color [9]. Our findings align with this perspective, as the expression of *VviMYBC2-L1* did not appear to be influenced by the miPEP166c treatment. This observation is likely due to the significant upregulation of transcript levels in both anthocyanin transcriptional activators (VviMYBA1 and VviMYBA2) and the proanthocyanidin transcriptional activator (VviMYBPA1) induced by miPEP166c. This supports the proposed theory that transcriptional activators may induce the upregulation of transcriptional repressors to maintain a balance in the synthesis of anthocyanins and proanthocyanidins [9]. Thus, a miR166c-mediated repression of *VviMYBC2-L1* was probably counterbalanced by a VviMYBA1/2-triggered increase in *VviMYBC2-L1* transcripts, and for that reason, no effect of miPEP166c on its transcript’s abundance was detected. However, the accentuated expression and availability of VvMYBA1/2 were per se sufficient to diminish the repressive action of VviMYBC2-L1 on the synthesis of proanthocyanidins and anthocyanins.

### 3.2. Exogenous miPEP166c Leads to Enhanced Synthesis of Anthocyanins and Proanthocyanidins in Gamay cv. Grape Berry Cells

By upregulating the expression of *VviMYBPA1* (Figure 4), the exogenous treatment with miPEP166c significantly induced the proanthocyanidin biosynthetic pathway, as observed by the transcriptional and biochemical changes in the key genes and enzymes that lead to the synthesis of flavan-3-ols (proanthocyanidin monomers). In this study, we also observed an upregulation in the transcripts of *VviDFR* and *VviLDOX*, which encode enzymes involved in earlier steps of the flavonoid pathway that synthesize important substrate molecules for both the anthocyanin and proanthocyanidin pathways. In accordance with the higher content of proanthocyanidins, the maximum velocities of VviLAR and VviANR activities were significantly increased by the application of miPEP166c to the cell cultures, and their respective genes were upregulated, as observed in Figure 5 and Figure 6.

The observed increase in the content of anthocyanins in miPEP166c-treated Gamay cells, together with the abundance of transcripts of both anthocyanin transcriptional activators (VviMYBA1 and VviMYBA2), was correlated with the increase in the expression levels of key anthocyanin gene *VviUFGT1*, accompanied by an increased enzyme activity of UFGT as well, as shown in Figure 9. This evidence demonstrates that the anthocyanin-synthetic pathway was stimulated by the exogenous addition of miPEP166c. Moreover, as demonstrated in Figure 10, anthocyanin stabilization and transport into the vacuole were also stimulated, as denoted by increased *VviGST4* and *VviMATE1* transcripts.

In sum, we have demonstrated that miPEP166c exogenous applications in Gamay Fréaux cv. grape berry cells significantly stimulated the anthocyanin and proanthocyanidin biosynthetic pathways by targeting VviMYBC2-L1 directly, diminishing its repressive action, and, for that reason, indirectly inducing the transcriptional activators VviMYBA1/2 and VviMYBPA1 in this complex regulatory network.

**Figure 11:**
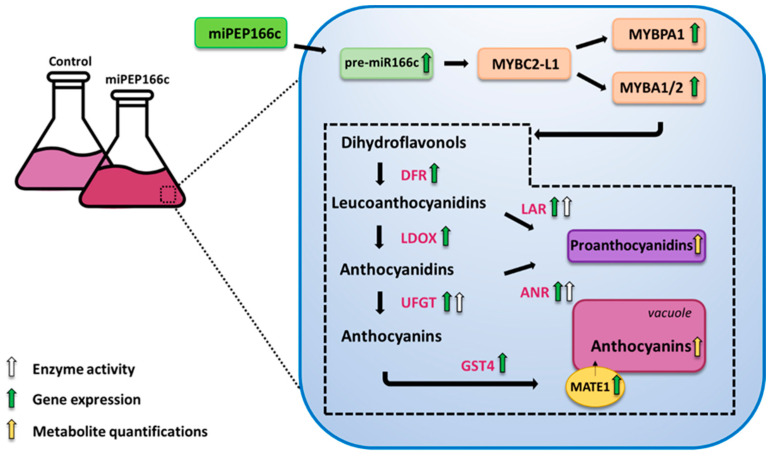
The exogenous application of miPEP166c increases anthocyanin and proanthocyanidin synthesis and accumulation by a miR166c-mediated downregulation of the transcriptional repressor MYBC2-L1 in Gamay Fréaux grape berry cell suspensions.

As illustrated in Figure 11, by increasing the transcript abundance of *pre-miR166c* and thus regulating the VviMYBC2-L1–VviMYBA1/2–VviMYBPA1 regulatory complex, miPEP166c enhanced the synthesis of anthocyanins and proanthocyanidins and their underlying molecular mechanisms.

## 4. Materials and Methods

### 4.1. Biological Materials

In this study, grape berry cell suspensions of *Vitis vinifera* L. cv. Gamay Fréaux cv. Tenturier were utilized. These cell suspensions were obtained and established through the dedifferentiation of grape berry mesocarp and were generously provided by Prof. Serge Delrot from the Institute of Vine and Wine Science (ISVV), University of Bordeaux. The grape berry cell suspensions were cultured in 250-mL flasks containing Gamborg B5 medium. The composition of the culture medium was as follows: 3 g/L Gamborg B5 salt mixture and vitamins, 30 g/L sucrose, 250 mg/L casein enzymatic hydrolysate, 0.1 mg/L α-napthaleneacetic acid (NAA), and 0.2 mg/L kinetin. The pH of the medium was maintained between 5.7 and 5.8. To provide optimal growth conditions, the flasks were placed in a controlled environment with a temperature of 23 °C. Constant agitation was applied using a rotator shaker set at 100 rpm. The cell suspensions were exposed to a 16 h light/8 h dark photoperiod, with a light intensity of 200 µmol photons m^2^/s. The light source wavelength was dominant at 450 nm (blue) and 620 nm (red), and the light source was a FLUORA OSRAM 36W/77 (14,000 lm) lamp. To sustain the cultures, sub-culturing was performed every 7 days, ensuring the continuous maintenance of the cell suspensions. This involved transferring a portion of the cells into fresh culture medium in new flasks to provide nutrients and maintain the growth of the cells. By following this cultivation protocol, the grape berry cell suspensions could be maintained and utilized for subsequent experiments in this study.

### 4.2. In Silico Analyses

To identify potential miPEPs targeting transcription factor VviMYBC2-L1, we conducted a series of in silico analyses by combining several bioinformatic tools and databases, such as the bioinformatic tool psRNATarget Finder [19], which was used to blast the sequence of *VviMYBC2-L1* to retrieve information on which previously described grapevine miRNAs could putatively regulate it. The identified mature miRNAs were then screened in miRBase (a microRNA database) [20] for their non-mature sequences of the regulatory miRNAs, possibly harboring small open reading frames (ORFs) corresponding to regulatory miPEPs. Finally, the obtained sequences were then run in a bioinformatic ORF finder tool that screens for putative ORFs that could translate into a small peptide by defining several parameters based on the few miPEPs identified in the literature so far [10,11,12,13,14]. This analysis matched our gene of interest with a known grapevine miRNA, miR166c, and resulted in a putative ORF of 44 bp coding a potential miPEP for its regulation. To study whether this miPEP was active in grape berry cell suspensions, we tested its exogenous application in vitro.

### 4.3. Solubilization of miRNA-Encoded Peptides

Upon the in silico identification of miPEP166c, the micropeptide, along with a scrambled version, was obtained as 1 mg aliquots from Smart Bioscience (www.sb-peptide.com, accessed on 8 July 2020). The solubilization process of these micropeptides followed the recommended guidelines provided by the Smart Bioscience Peptide Solubility Guidelines. To solubilize the micropeptides, each aliquot was dissolved in 1 mL of pure water, resulting in a final concentration of 1 mg/mL. Subsequently, the solubilized micropeptides were sterilized through filtration to ensure their purity before further use in the experiments. The specific sequences of miPEP166c and scrambled miPEP166c were as follows: miPEP166c: MLSTNKNTIIHIYR, and scrambled miPEP166c: NIILTRNMTHIYSK. These solubilized micropeptide solutions were then ready for application and further investigation in the subsequent experimental procedures.

### 4.4. Exogenous Application of Micropeptides to Gamay Grape Cells

Following each sub-cultivation, cell cultures received the addition of miPEP166c solubilized in pure water at concentrations of 0.1 µM, 0.5 µM, and 1 µM. The cell suspensions, which included control cells treated with an equivalent volume of the control solution as well as cells treated with the scrambled version of miPEP, underwent a 10-day cultivation period with continuous agitation and a 16 h light/8 h dark photoperiod. Subsequently, the cells were harvested, filtered using a vacuum filtration apparatus, promptly frozen using liquid nitrogen, and stored at −80 °C. Cells were then freeze-dried for 3 days to remove water content and used as a fine powder in all experiments.

### 4.5. Quantification of Anthocyanins

Anthocyanins were assessed using the method outlined by Conde et al. [21]. Extraction involved utilizing 100 mg of grape berry cells from each experimental condition. Upon the addition of 1 mL of 90% methanol, the suspensions underwent vigorous shaking in an orbital shaker for 30 min at 200 rev/min, followed by centrifugation at 18,000× *g* for 20 min. The resulting supernatants were collected, and 100 μL of each was combined with 900 µL of a 25 mM KCl solution (pH = 1.0). Absorbance readings at 520 nm and 700 nm were obtained using a Shimadzu UV-160A spectrophotometer (Kyoto, Japan). The quantification of total anthocyanins was determined in relation to cyanidin-3-glucoside equivalents, as follows:

$$\mathrm{Total}\;\mathrm{anthocyanins}\;\left(\mathrm{mg}/g\;\mathrm{DW}\right)=\;\frac{(A_{520}-A_{700})\;\times \;\mathrm{MW}\;\times \;\mathrm{DF}\;\times \;1000}{\varepsilon \;\times \;1}$$

where MW represents the molecular weight of cyanidin-3-glucoside (449.2 g mol^−1^), DF stands for the dilution factor, and ε denotes the molar extinction coefficient of cyanidin-3-glucoside (26,900 M^−1^ cm^−1^). The concentration was then expressed per milligram of dry weight (DW) of the utilized cells.

### 4.6. Quantification of Proanthocyanidins

Proanthocyanidin levels were assessed through the DMACA assay, as refined by Wallace and Giusti [22]. For proanthocyanidin extraction, 100 mg of frozen grape berry cells were treated with 1 mL of 90% methanol, vigorously shaken for 30 min in an orbital shaker at 200 rev/min, and then subjected to centrifugation at 18,000× *g* for 15 min. The DMAC reagent (4-dimethylaminocinnamaldehyde) was freshly prepared by dissolving 2% of DMAC reagent (*w/v*) in a 1:1 mixture of methanol and 6N H_2_SO_4_ (*v/v*). This solution was kept on ice and shielded in aluminum foil to protect it from the light sensitivity inherent to DMAC. The assay mixture comprised 1.175 mL of methanol and 50 µL of DMAC (2%), and the reaction was initiated by adding 10 µL of each experimental condition. Following a 15-min reaction at room temperature under subdued light conditions, absorbance was measured at 640 nm using a Shimadzu UV-160A spectrophotometer (Kyoto, Japan). A standard curve using (+)-catechin and DMAC, with concentrations ranging from 50 to 500 µg/mL, was freshly prepared for each proanthocyanidin quantification. The results were expressed as catechin equivalents per gram of dry weight (DW).

### 4.7. Protein Extraction

The extraction of proteins was carried out following the procedure outlined by Badim et al. [23]. Grape berry cell powder was combined with extraction buffer at an approximate ratio of 1:2 (*v/v*) powder to buffer. The protein extraction buffer comprised 50 mM Tris-HCl (pH 8.9), 5 mM MgCl2, 1 mM EDTA, 1 mM phenylmethylsulphonyl fluoride (PMSF), 5 mM dithiothreitol (DTT), 0.1% (*v/v*) Triton X-100, and 1% (*w/v*) polyvinylpolypyrrolidone (PVPP). The homogenates were vortexed for 20 min and then centrifuged at 18,000× *g* for 20 min at 4 °C. The resulting supernatants were kept on ice and utilized for all enzymatic assays. The total protein concentrations in the extracts were determined using the Bradford method [24], with bovine serum albumin as the standard.

### 4.8. Enzymatic Activity Assays

The enzymatic activity of UDP-glucose: flavonoid 3-O glucosyltransferase (UFGT) was determined following the protocol described by Lister et al. [25] with adaptations by Conde et al. [21]. The assay mixture included a 300 mM Tris-HCl reaction buffer (pH 8), 200 µL of enzyme extract, and 1 mM UDP-glucose. The reaction was initiated with 1 mM quercetin as a substrate for enzyme activity (saturating concentration), reaching a final reaction volume of 500 µL. Each mixture underwent a 30-min incubation in the dark at room temperature with gentle shaking using an orbital shaker. Dilutions were prepared before and after the incubation period by combining 100 µL of each assay mixture with 900 µL of Tris-HCl reaction buffer, and absorbance was read at 350 nm immediately after (t = 0) and 30 min later (t = 30) to monitor the production of quercetin 3-glucoside (ε = 21,877 M^−1^ cm^−1^).

Leucoanthocyanidin reductase (LAR) enzymatic activity was assessed by spectrophotometrically tracking the conversion of dihydroquercetin into (+)-catechin following the method outlined by Gagné et al. [26] with some modifications. The assay mixture comprised 1.7 mL of Tris-HCl buffer (0.1 M, pH 7.5), 300 µL of crude protein extract, and 2 µL of NADPH (100 mM), and the reaction was initiated by adding 1 µL of dihydroquercetin (10 mg mL^−1^ in DMSO). The production of (+)-catechin (ε = 10,233 M^−1^ cm^−1^) was monitored at 280 nm for 30 min following the increase in absorbance of the reaction mixture.

The enzymatic activity of anthocyanidin reductase (ANR) was determined according to the protocol by Zhang et al. [27]. The 1.5 mL assay mixture included 0.1 M PBS buffer (pH 6.5), 100 µL of enzyme extract, 1 mM ascorbic acid, and 0.07 mM cyanidin chloride. The reaction was initiated by adding 1 mM NADPH, followed by a 1/10 dilution with PBS reaction buffer for proper absorbance measurement. The enzyme activity was monitored by measuring the rate of NADPH oxidation (ε = 6.22 mM^−1^ cm^−1^) at 340 nm for 20 min at 45 °C. A control experiment was conducted under the same conditions without adding cyanidin chloride. All enzyme activities were measured using a Shimadzu UV-160A spectrophotometer (Kyoto, Japan).

### 4.9. RNA Extraction and cDNA Synthesis

RNA extraction was conducted following the methodology outlined by Reid et al. [28], with additional purification steps incorporated from the GRS Total Plant RNA extraction kit (Grisp, Porto, Portugal). Approximately 200 mg of fresh cells treated with miPEP166c and control cells were employed for RNA extraction using a modified extraction buffer containing 2% (*w/v*) CTAB, 2% (*w/v*) PVP K-30, 300 mM Tris-HCl (pH 8.0), 25 mM EDTA, 2 M NaCl, and 40 mM DTT. Following in-column treatment with DNase I (Thermo Scientific^TM,^, Waltham, MA, USA), cDNA was synthesized from 1 µg of total RNA using the Xpert cDNA Synthesis Master-mix Kit (Grisp, Porto, Portugal), following the manufacturer’s instructions. The concentration and purity of RNA were determined using Nanodrop, and its integrity was assessed on a 1% agarose gel stained with SYBR Safe (Invitrogen^TM^, Life Technologies, Waltham, MA, USA).

### 4.10. Transcriptional Analyses through Real-Time qPCR

Quantitative real-time PCR was conducted utilizing Xpert Fast SYBR Blue (Grisp, Porto, Portugal) on a CFX96 Real-Time Detection System (Bio-Rad, Algés, Portugal). Each well contained 1 µL of cDNA in a final reaction volume of 10 µL. The specific primer pairs employed for each target gene are detailed in Table S1. Melting curve analysis was executed to confirm the specificity of gene amplification. *VvACT1* (actin) and *VvGAPDH* (glyceraldehyde-3-phosphate dehydrogenase) were chosen as reference genes, given their established stability and suitability for qPCR normalization in grapevine [25]. Two independent runs with triplicates were performed for all experimental conditions tested. Expression values were normalized by the average expression of the reference genes, following the methodology described by Pfaffl [29], and data analysis was carried out using the Bio-Rad CFX Manager software 3.1 (Bio-Rad, Algés, Portugal).

### 4.11. Statistical Analyses

Statistical analysis of the results was conducted using the Student’s *t*-test in Prism 9 (GraphPad Software, Inc., Boston, MA, USA). The data are expressed as the mean ± standard deviation of the mean (SD), and statistical comparisons were made with a 95% confidence interval. Significance levels between the mean values for each condition are indicated by asterisks based on the *p*-values, with * denoting *p* < 0.05, ** for *p* < 0.01, *** for *p* < 0.001, and **** for *p* < 0.0001. Each experimental approach utilized a minimum of three biological replicates and was repeated three times.

## Figures and Tables

**Table 1:** Detailed information about miPEP166c including its corresponding mature miRNA and mode of action.

miPEP Name	Amino Acid Sequence	miRNA Name	Stem–Loop Sequence	Mode of Action	Predicted Target
miPEP166c	MLSTNKNTIIHIYR	miR166c (MIMAT0005664)	MI006509	Inhibition in cleavage	*VviMYBC2-L1*

## Data Availability

All data generated or analyzed during this study are included in this article. Further enquiries can be directed to the corresponding author.

## Supplementary Materials

The supporting information can be downloaded at: https://www.mdpi.com/article/10.3390/ijms25031539/s1. References [2,4,5,30,31,32,33,34,35] are cited in the Supplementary Material
