# Supplementary material for: Non-Mature miRNA-Encoded Micropeptide miPEP166c Stimulates Anthocyanin and Proanthocyanidin Synthesis in Grape Berry Cells
Source: Int J Mol Sci. 2024 Jan 26;25(3):1539. doi: 10.3390/ijms25031539 (PMC10855927; doi:10.3390/ijms25031539)
Supplement: Supplementary file 1 [file ijms-25-01539-s001.zip › ijms-2809856-supplementary.pdf]

Supplementary Table S1: Primers forward (F) and reverse (R) used for gene expression analysis by qPCR.

Gene	Acession number	Primers	Ref.
VviDFR	Vitvi18g00988	F: 5′-GGCTTTCTAGCGAGAGCGTA-3′<br>R: 5′-ACTCTCATTTCCGGCACATT-3′	[30]
VviLDOX	Vitvi13g00055	F: 5′-ACCTTCATCCTCCACAACAT – 3′<br>R: 5′-AGTAGAGCCTCCTGGGTCTT – 3′	[31]
VviLAR1	Vitvi01g00234	F: 5′-CAGGAGGCTATGGAGAAGATAC – 3′<br>R: 5′-ACGCTTCTCTCTGTACATGTTG – 3′	[31]
VviANR	Vitvi10g02185	F: 5′-CAATACCAGTGTTCCTGAGC – 3′<br>R: 5′-AAACTGAACCCCTCTTTCAC – 3′	[31]
VviUFGT1	Vitvi16g00156	F: 5′-TGCAGGGCCTAACTCACTCT-3′<br>R: 5′-GCAGTCGCCTTAGGTAGCAC-3′	Designed with the aid of QuantiPrime
VviGST4	Vitvi04g00880	F: 5′-AAGGATCCATGGTGATGAAGGTGTATGGC-3'<br>R: 5′-AACTGCAGAAGCCAACCAACCAACAAAC-3′	[32]
VviMATE1	Vitvi16g01911	F: 5′-TGCTTTTGTGATTTTGTTAGAGG-3′<br>R: 5′-CCCTTCCCCGATTGAGAGTA-3′	[33]
VviMYBPA1	Vitvi13g00055	F: 5′-AGATCAACTGGTTATGCTTGCT-3<br>R: 5′-AACACAAATGTACATCGCACAC-3	[2]
VviMYBA1	Vitvi02g01019	F: 5′-AGAACAGGTTCGAGGTTG-3′<br>R: 5′-TCTATTCAACCCTGCTCGG-3	[5]
VviMYBA2	Vitvi02g01015	F: 5′-GCAGGGTTGAATAGATGCCTAAA-3′<br>R: 5′-CTCGTCTAATGCAAACTCTCCTCTC-3′	[34]
VviMYBC2-L1	Vitvi01g00401	F: 5′ -GCAACCGCTGGTCACTGAT-3′<br>R: 5′ -CGAGGGCGAGGGAAATTGT-3′	[4]
Vvipre-miR166c	NR_127751	F: 5′-TTGAGCAAGATGGAGAAGCA – 3′<br>R: 5′-ATTGGTTTGTGGTGCATGAG – 3′	Designed in this study
VviActin	Vitvi04g01613	F: 5′-GTGCCTGCCATGTATGTTGCC-3′<br>F: 5′-GTGCCTGCCATGTATGTTGCC-3′	[35]
VviGADPH	GSVIVT00009717001	F: 5′-CACGGTCAGTGGAAGCATCAT-3′<br>R: 5′-CCTTGTCAGTGAACACACCAG-3′	[35]
